# Poor Results of Flow Diversion as Salvage Treatment for Intracranial Aneurysm Rerupture After Surgical Clip Reconstruction

**DOI:** 10.7759/cureus.6137

**Published:** 2019-11-12

**Authors:** Craig J Kilburg, Min S Park, Yashar Kalani, Philipp Taussky

**Affiliations:** 1 Neurosurgery, Baptist Health, Jacksonville, USA; 2 Neurosurgery, University of Virginia, Charlottesville, USA; 3 Neurosurgery, Barrow Neurological Institute, Charlottesville, USA; 4 Neurosurgery, University of Utah School of Medicine, Salt Lake City, USA

**Keywords:** flow diverter, pipeline embolization device, rerupture, ruptured aneurysm, salvage surgery

## Abstract

Rebleeding episodes after a ruptured intracranial aneurysm has been secured are considered a significant source of patient morbidity and mortality. Theoretically, acute treatment with a flow-diversion device may offer a reasonable treatment option to prevent future bleeding and to remodel the diseased vessel segment. The authors identified two patients who underwent emergent treatment with the placement of a Pipeline Embolization Device (PED) in the setting of an acute rebleeding of a ruptured intracranial aneurysm previously treated with clip reconstruction. The first patient was a 50-year-old woman who underwent clip reconstruction for a broad-based right anterior choroidal artery aneurysm measuring approximately 2×8 mm. Clip reconstruction was achieved with a single fenestrated clip. On day 14, the patient experienced a rebleeding episode. She underwent emergent treatment with a single PED but experienced another rebleeding and died. The second patient was a 53-year-old woman who presented with a ruptured dorsal variant blister aneurysm, which was treated with clip reconstruction. On day 22, she experienced a rebleeding episode and underwent emergent treatment using two PEDs in a duplicative fashion. After the procedure, she experienced another acute rebleeding episode and died. The treatment of reruptured intracranial aneurysms in a salvage fashion with emergent placement of PEDs in two patients resulted in good technical placement of the device covering the neck of the aneurysm, yet both patients experienced additional rebleeding and did not survive. Future generations of flow diverters may have more appropriate properties that would allow their use as salvage treatment in the setting of acutely ruptured aneurysms.

## Introduction

Intracranial aneurysm rerupture after surgical or endovascular treatment is a catastrophic complication and a major cause of patient morbidity and mortality [1-4]. Several large studies have shown the overall rate of early aneurysm rerupture after treatment to be between 0.9% and 5% [1-6]. Once a rebleeding occurs, the overall mortality rate has been determined to range from 46% to 100% [1-4].

No consensus exists regarding the management of patients who experience a rebleeding episode. Surgical decision making is often made more complex by the poor clinical state of patients and the significant risk and technical challenges of a reoperation. We present two cases of ruptured paraclinoidal aneurysms that were initially treated with clip reconstruction; each patient experienced a delayed rebleeding episode and underwent salvage treatment with Pipeline flow diversion (Medtronic, Plymouth, MN). From a technical perspective, the attempts at management were successful, but without clinical success.

## Case presentation

Case 1

**Figure 1:**
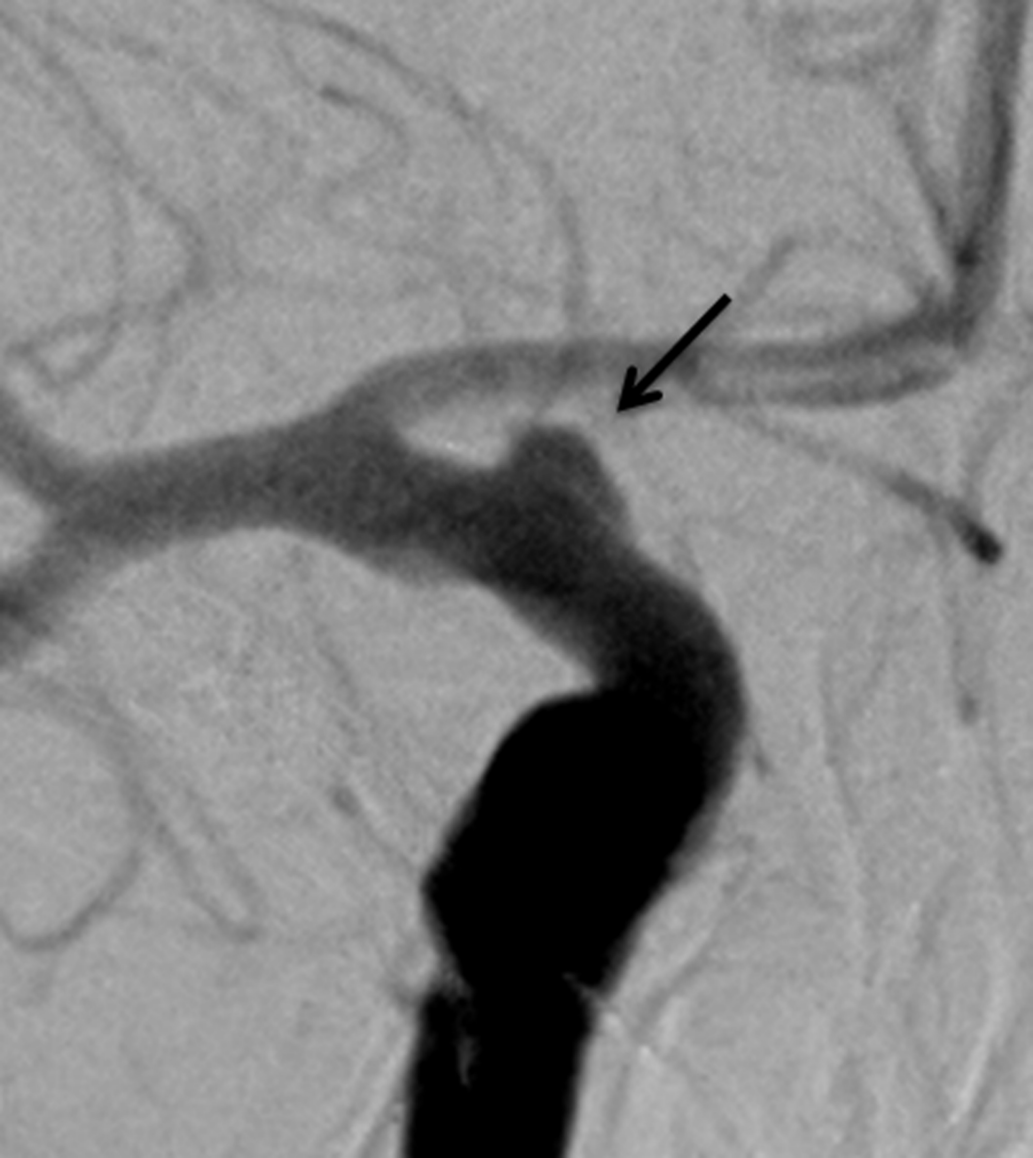
Case 1. Digital subtraction angiography demonstrating a broad-based right anterior choroidal aneurysm (arrow).

**Figure 2:**
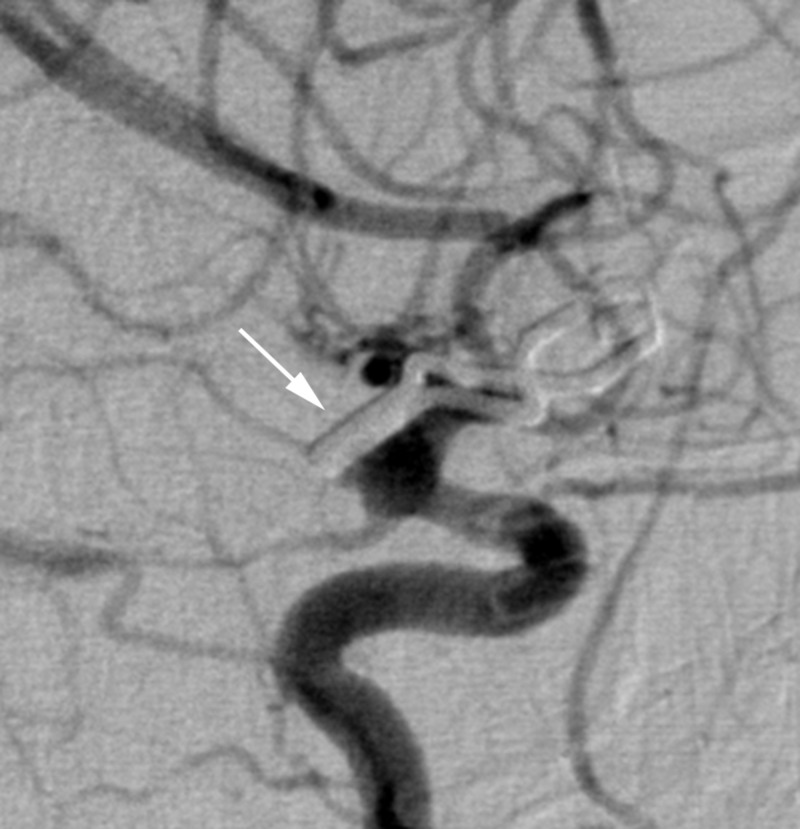
Case 1. Digital subtraction angiography from hospital day 8 demonstrating complete obliteration of the aneurysm (arrow).

A 50-year-old woman with a personal history of hypertension and a family history of intracranial aneurysms presented initially to an outside hospital with sudden-onset severe headache, nausea, vomiting, and dizziness (Hunt Hess 2, Fisher grade 3). A computed tomography angiography (CTA) scan of the head and digital subtraction angiography (DSA) (Figure 1) demonstrated a broad-based right anterior choroidal artery aneurysm measuring approximately 2×8 mm. She was subsequently transferred to our hospital, and a right frontotemporal craniotomy was performed with successful clip reconstruction of the aneurysm with a single fenestrated clip. On hospital day 8, a surveillance DSA demonstrated evidence of complete obliteration of the aneurysm and mild anterior circulation vasospasm, right greater than left, that was treated with an intra-arterial infusion of verapamil (Figure 2).

**Figure 3:**
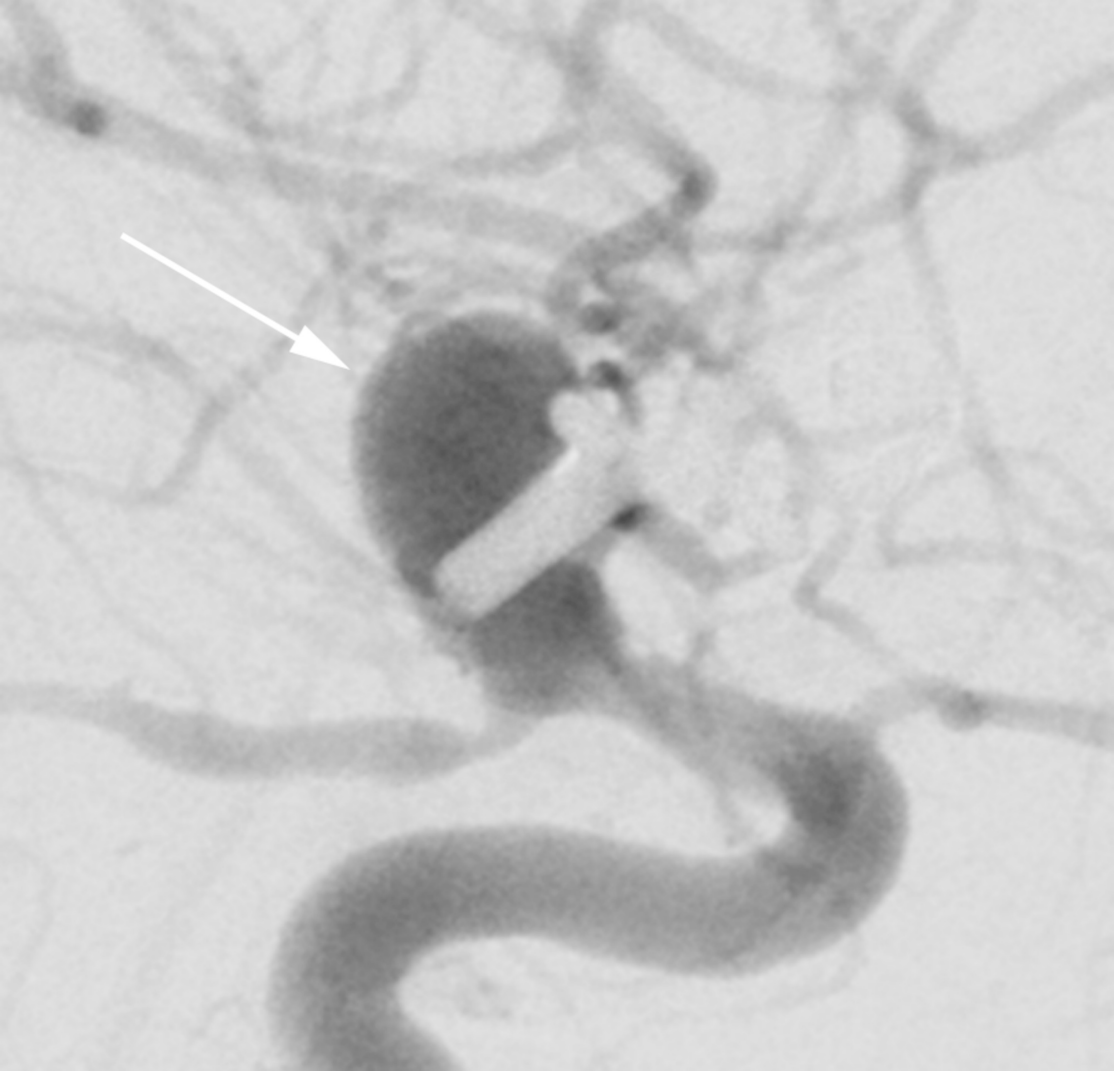
Case 1. Digital subtraction angiography from hospital day 14 immediately prior to flow diverter placement demonstrating recurrence of the aneurysm (arrow).

On hospital day 14, the patient had an acute worsening of her headache followed by a rapid decline in her neurologic examination until she exhibited only extensor posturing to noxious stimuli (Hunt Hess 5). A follow-up CTA demonstrated concern for recurrence of her aneurysm with evidence of new subarachnoid hemorrhage concerning for repeat aneurysm rupture. An emergent DSA confirmed a recurrence of the aneurysm, which was projected superiorly from the clip site and measured approximately 8 mm in its maximal dimensions (Figure 3). The recurrence was thought to represent a pseudoaneurysm, and as a result, it was thought that the patient would be a high-risk candidate for either repeat surgical clip reconstruction or coil embolization. After discussion with the patient’s family, the decision was made to treat the recurrence with flow diversion.

**Figure 4:**
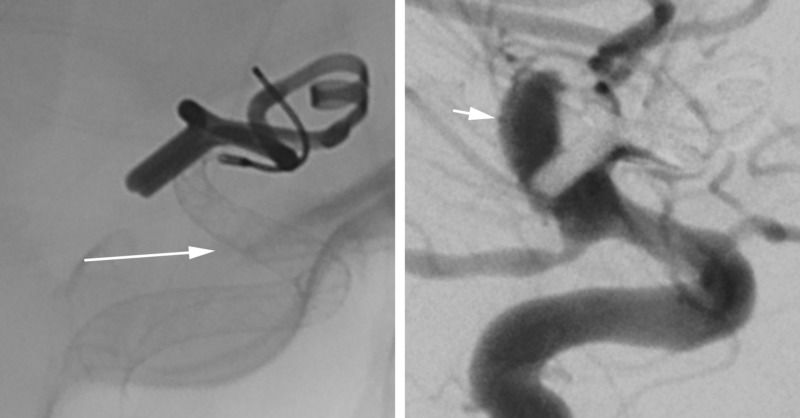
Case 1. Unsubtracted (left) and subtracted (right) views from the same angiographic sequence immediately following device placement demonstrating complete coverage of the aneurysm neck by the device (arrow) and delayed filling of and contrast stasis within the aneurysm (arrowhead).

The patient was given 20 mg of intravenous abciximab and underwent uncomplicated placement of a single 4×20 mm Pipeline Embolization Device. Immediate follow-up angiograms after deployment demonstrated contrast stasis within the recurrence (Figure 4). At the time of the angiogram, the patient was also noted to have severe anterior circulation vasospasm bilaterally that was treated with an intra-arterial infusion of verapamil. Blood pressures were kept below 160 mm Hg systolic due to concern for aneurysm rerupture, despite the presence of vasospasm. After the procedure, she remained intubated with severely depressed neurologic examination findings. Repeat cerebral angiogram the next day showed continued severe anterior circulation vasospasm that was treated with intra-arterial verapamil and angioplasty of the left middle cerebral artery. Later that night, she had an acute elevation of her intracranial pressure to >90 mm Hg and further decline of her neurologic examination. Aggressive medical management was initiated including boluses of sedation, hypertonic saline, and mannitol. Her intracranial pressure improved, but remained elevated at approximately 30 mm Hg. A repeat CT scan of the head demonstrated extensive new intraventricular hemorrhage concerning for repeat aneurysm rupture. Shortly after the scan, the patient developed ventricular tachycardia, experienced cardiac arrest, and died.

Case 2

**Figure 5:**
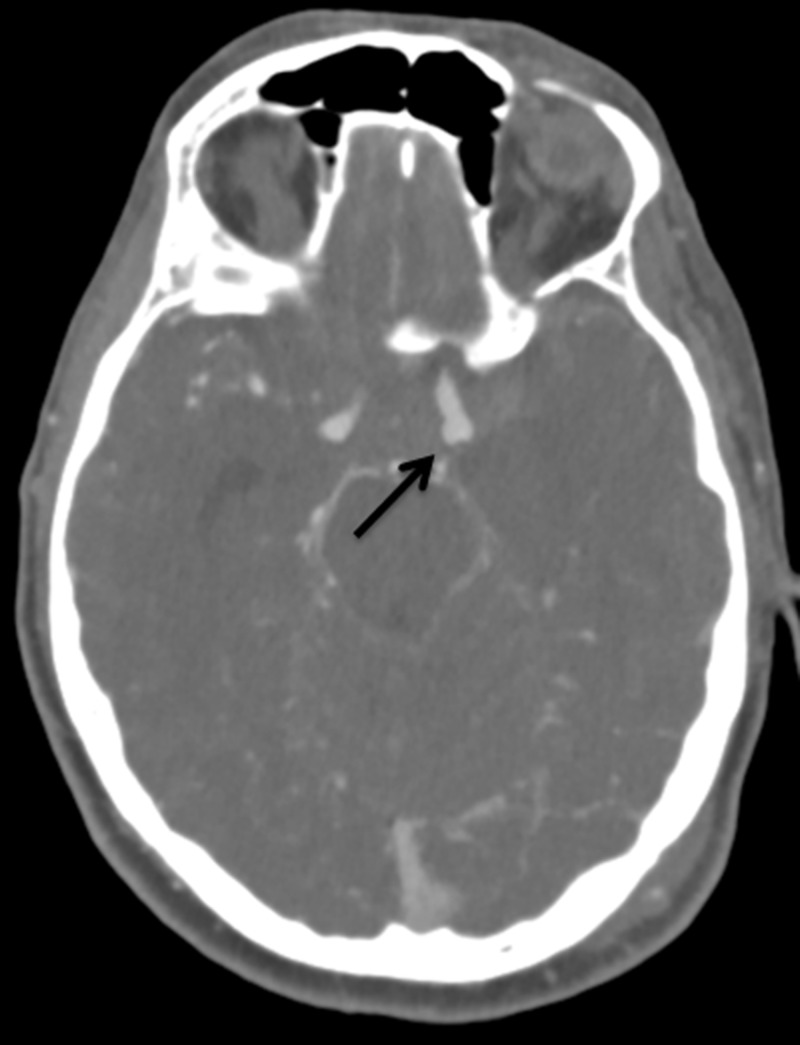
Case 2. Preoperative computed tomography angiography demonstrating the left internal carotid artery dorsal variant aneurysm (arrow).

A 53-year-old woman with no significant past medical history presented initially to an outside hospital with a two-day history of a mild headache followed by acute worsening of the headache, nausea, vomiting, and loss of consciousness immediately prior to presentation. A CT scan of the head demonstrated diffuse, thick subarachnoid hemorrhage, a small amount of intraventricular hemorrhage, and an intraparenchymal hemorrhage within the inferior left frontal lobe (Fisher grade 4). The patient was then transferred to our hospital for definitive management. Upon arrival at our hospital, she was intubated, stuporous, localizing to noxious stimuli in her left arm, and withdrawing to noxious stimuli in her right arm and legs (Hunt Hess 4). A CTA demonstrated a dorsal variant aneurysm of the left internal carotid artery (Figure 5). The patient was taken to the operating room that day for a left frontotemporal craniotomy for clip reconstruction of the aneurysm with a single fenestrated clip. It was felt during surgery that the vessel could be successfully reconstructed using clip reconstruction without clip wrapping.

**Figure 6:**
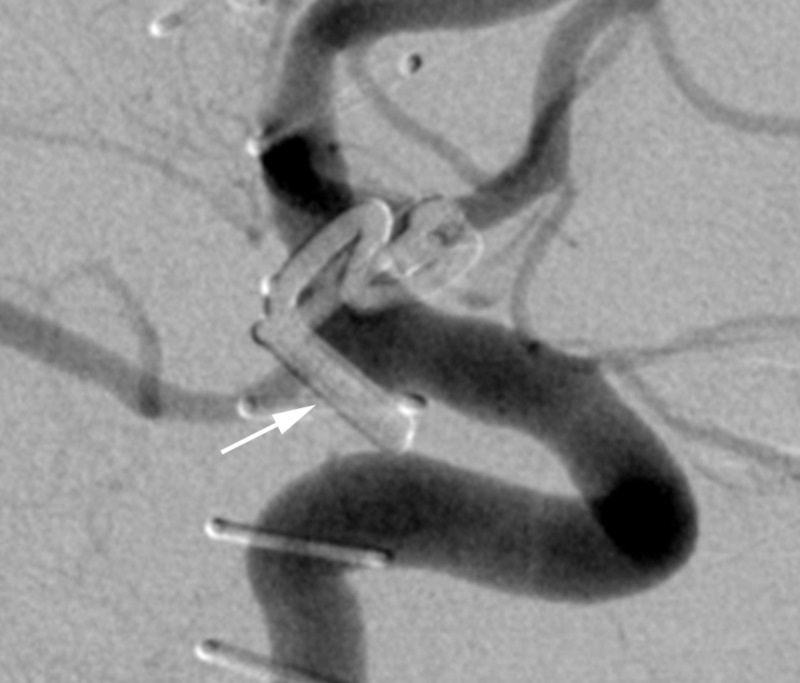
Case 2. Digital subtraction angiography from hospital day 3 demonstrating complete obliteration of the aneurysm (arrow).

**Figure 7:**
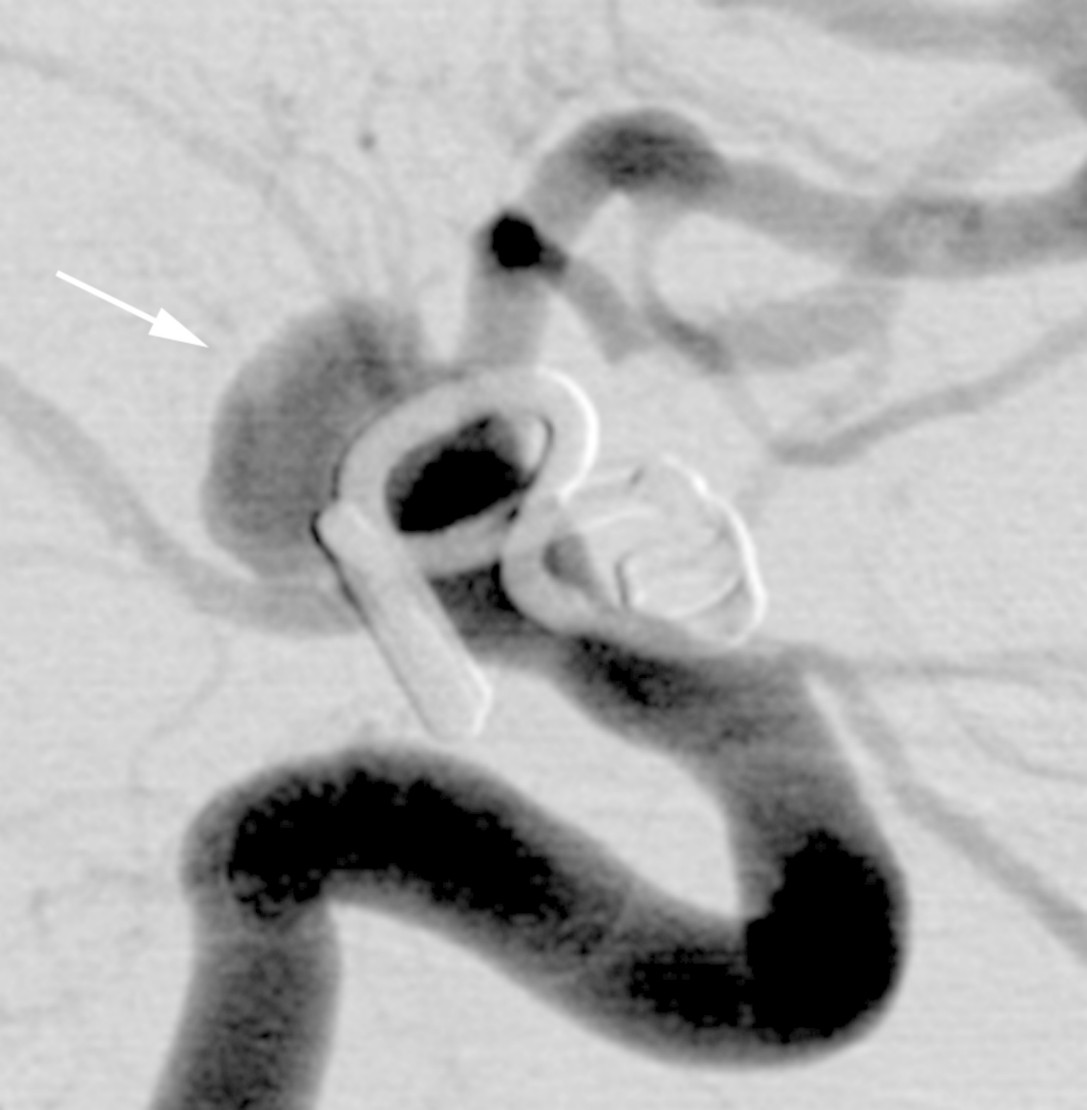
Case 2. Digital subtraction angiography from hospital day 22 immediately prior to device placement demonstrating distal recurrence of the aneurysm (arrow).

**Figure 8:**
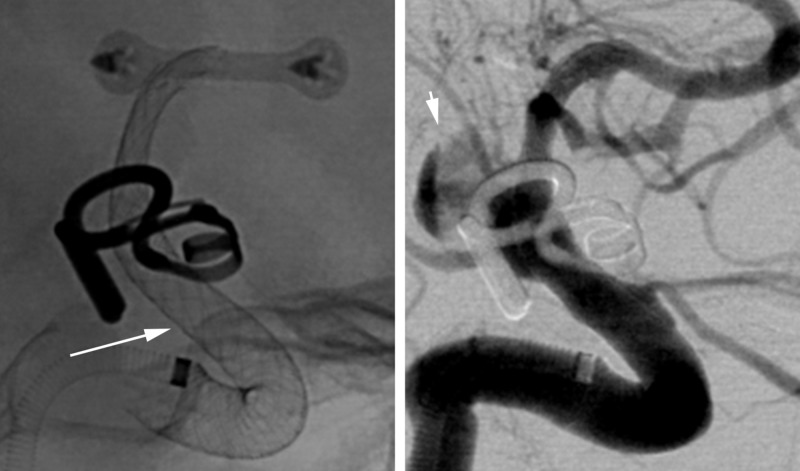
Case 2. Unsubtracted (left) and subtracted (right) views from the same angiographic sequence immediately after placement of the second device demonstrating complete coverage of the aneurysm neck by the device (arrow) and delayed filling of the aneurysm (arrowhead).

Postoperatively, her neurologic examination findings remained depressed but consistent with her examination at presentation. On hospital day 3, a surveillance DSA demonstrated reconstruction of the vessel without evidence of residual aneurysm filling or significant vasospasm (Figure 6). The patient’s clinical examination slowly improved over the next several days. On hospital day 21, an intravenous heparin infusion was started for several persisting deep venous popliteal clots. On hospital day 22, the patient became acutely somnolent. A CT scan of the head demonstrated new subarachnoid hemorrhage around the clip site and a new, thin subdural hematoma along the left convexity concerning for aneurysm rerupture. An emergent DSA showed a new dorsal variant aneurysm just distal to the previous clip site (Figure 7). The new aneurysm was multilobulated and measured approximately 7 mm in its maximal dimensions. There was also evidence of active pulsations within the aneurysm during angiography. The collective decision of the patient’s family, and the treating neurosurgeon was to proceed with treatment by flow diversion. The patient was given 10 mg of intravenous abciximab, and a 4.5×25 mm Pipeline Flex device (ev3/Covidien/Medtronic) was placed across the aneurysm. The patient was then given a second dose of 10 mg of abciximab. To provide as much protection as possible, the decision was made to cover the aneurysm with a second flow diverter. Another 4.5×20 mm Pipeline Flex device was then placed within the first device in a duplicative fashion. An immediate follow-up angiogram demonstrated good contrast stasis within the aneurysm (Figure 8).

Immediately after the procedure, the patient was taken for a repeat CT scan, which demonstrated new, diffuse subarachnoid hemorrhage and intraventricular hemorrhage, which was expected due to her prior decline in her neurological status. On examination, her pupils were nonreactive bilaterally and she was not responding to noxious stimuli. She also required a vasopressor infusion to maintain her hemodynamic function. The patient’s family decided to withdraw care, and the patient died.

## Discussion

A rerupture of a previously treated ruptured intracranial aneurysm is not only a source of significant morbidity and mortality, with a reported mortality rate of 46%-100% [1-4], but it is a true surgical emergency with often limited options and no consensus on best treatment. Patients are often in a very poor clinical state, and in such instances, one of the main questions is whether treatment should be offered at all in the face of an almost certainly fatal outcome; however, not all reruptures are immediately catastrophic, and there should be a strong consideration for resecuring the aneurysm. Surgical options tend to involve performing a large hemicraniectomy to help with the management of intracranial pressure and attempts at either clip wrapping the aneurysm, or trapping the diseased segment with or without bypass. However, salvage treatment often does not allow surgical exploration because of the clinical status of the patient, high intracranial pressure, and the concern that in the presence of failed microsurgical clipping an alternative treatment option should be used [7,8]. In these cases, endovascular treatment may be a more clinically reasonable option.

In the two cases presented here, rebleeding occurred because of new aneurysm formation after initial successful clip reconstruction and occlusion. In cases like these, the rapid formation and rupture of a new aneurysm indicates that the diseased segment of the vessel needs to be reinforced. As such, flow diversion poses a theoretical optimal treatment alternative, although the presence of a ruptured aneurysm makes using this treatment strategy a difficult choice. One of the strengths of flow diversion is its ability to remodel a diseased segment, thus offering a true cure of the diseased segment and its aneurysm [8-10]. Moreover, in the two cases presented here, because of the rapid formation and hemorrhage of these aneurysms, there was concern that these manifested pseudoaneurysms, in which case, coiling is not a feasible endovascular treatment option, leaving only microsurgery or flow diversion as potential treatment modalities.

Flow diversion for salvage treatment is a potentially attractive option because it can often be employed regardless of what initial treatment was used. Several studies have analyzed the use of flow diversion as adjunctive treatment of unruptured aneurysms that failed initial treatment [8,10,11]. Moreover, flow diversion has been shown to be effective in acutely ruptured blister aneurysms [12], although we have recently reported a case of rerupture of a blister aneurysm after treatment with a single Pipeline device [13].

Although the cases presented here were technically successful, we believe they raise important questions regarding the management of reruptured aneurysms in the future, specifically whether the use of Pipeline flow diverters can be effective in preventing further hemorrhages from these unstable aneurysms. Because of our experience, we now treat all ruptured pseudoaneurysms and true aneurysms with at least two Pipeline flow diverter devices, even though our second patient most likely experienced a fatal rerupture after the treatment with two Pipeline devices. Because flow diversion does not lead to immediate aneurysm occlusion, salvage therapy of actively growing and hemorrhaging aneurysms, such as was presented here, may not be suitable for flow diversion.

Finally, the hemorrhagic and thrombotic risks associated with flow-diversion devices present additional concerns regarding their use in salvage situations. Initiating the dual-antiplatelet therapy required for their usage is often complicated because pretreating patients with aspirin and clopidogrel is often not possible as a result of the emergent nature of these cases. Pretreating with prasugrel rather than clopidogrel may be an option because it has a rapid therapeutic onset of 30-60 minutes; however, GPIIa/IIIb inhibitors are often chosen instead because of their immediate and controlled intraoperative application. Madaelil et al. [14] showed that the risk of thromboembolic complications during flow diverter placement for ruptured aneurysms was actually relatively low at 5% (5/111); however, the use of dual-antiplatelet therapy in the setting of an acutely ruptured aneurysm remains controversial because of the overall increased hemorrhagic risk associated with its use in intensive care unit settings.

## Conclusions

Although we believe that future generations of flow diverters may have more appropriate properties that would allow their use for the treatment of acutely reruptured aneurysms, neither of the patients in our experience survived despite technically successful placement. We believe that our two cases offer valuable insight into the dilemmas associated with the use of Pipeline flow diverters as salvage therapy for acutely reruptured aneurysms. Because flow diversion does not lead to immediate aneurysm occlusion, salvage therapy of actively growing and hemorrhaging aneurysms, such as was presented here, may not be suitable for flow diversion. 
